# Adaptive Surgical Decision-Making for Synchronous Metastatic Pancreatic Cancer and Early Breast Cancer with a BRCA1 Pathogenic Variant: A Case Report

**DOI:** 10.70352/scrj.cr.26-0257

**Published:** 2026-08-15

**Authors:** Takahiro Akahori, Tomoyo Yokotani, Akihiko Yoshizawa, Tomoko Uchiyama, Ryo Yamaguchi, Masayuki Sho

**Affiliations:** 1 Department of Surgery, Nara Medical University, Kashihara, Nara, Japan; 2 Department of Diagnostic Pathology, Nara Medical University, Kashihara, Nara, Japan

**Keywords:** breast neoplasms, pancreatic neoplasms, BRCA1 protein, neoplasms, multiple primary, poly(ADP-ribose) polymerase inhibitors

## Abstract

**INTRODUCTION:**

Synchronous pancreatic and breast cancers associated with a breast cancer susceptibility gene (BRCA)1 pathogenic variant are extremely rare. When 1 malignancy presents as stage IV disease, determining the optimal timing and extent of surgical intervention for a second primary cancer represents a major clinical challenge.

**CASE PRESENTATION:**

A woman in her 60s was diagnosed with synchronous stage IV pancreatic tail cancer with multiple liver metastases and stage I left breast cancer. S-1/oxaliplatin/irinotecan (SOXIRI) was initiated for metastatic pancreatic cancer, achieving a sustained partial response. Germline testing revealed a BRCA1 pathogenic variant. Maintenance olaparib was subsequently introduced, resulting in durable disease stabilization and normalization of tumor markers. While the pancreatic cancer remained controlled, the breast cancer progressed with axillary lymph node involvement. After confirming long-term systemic stability and preserved performance status, breast-conserving surgery with level II axillary lymph node dissection was performed 28 months after treatment initiation. At 77 months, the patient remains alive without breast cancer recurrence or pancreatic cancer progression.

**CONCLUSIONS:**

Adaptive surgical decision-making for a concurrent operable malignancy, supported by durable systemic control of a dominant stage IV cancer, may help prevent the malignancy from becoming an additional threat to long-term prognosis. Dynamic reassessment of oncologic priority is essential in the era of molecularly targeted therapy.

## Abbreviations

BRCA
: breast cancer susceptibility gene
CA19-9
: carbohydrate antigen 19-9
CEA
: carcinoembryonic antigen
ER
: estrogen receptor
HRD
: homologous recombination deficiency
IRIS
: irinotecan and S-1
PARP
: poly (ADP-ribose) polymerase
SEER
: Surveillance, Epidemiology, and End Results
SOXIRI
: S-1/oxaliplatin/irinotecan

## INTRODUCTION

Hereditary breast and ovarian cancer syndrome caused by germline pathogenic variants in breast cancer susceptibility genes BRCA1 or BRCA2 increases the lifetime risk of breast, ovarian, and pancreatic cancers.^1,2)^ Platinum-based chemotherapy and PARP inhibitors have improved outcomes in selected patients with BRCA-associated metastatic pancreatic cancer.^3,4)^ Olaparib has also demonstrated efficacy in germline BRCA-mutated metastatic breast cancer.^5)^ However, when synchronous BRCA-associated malignancies include stage IV disease, there are no established guidelines for treatment prioritization, optimal timing of surgical intervention for the concurrent early breast cancer, or sequential coordination of targeted systemic therapies. We report long-term survival in a patient with synchronous stage IV pancreatic cancer and early breast cancer, both associated with a germline BRCA1 pathogenic variant, managed through an adaptive treatment strategy incorporating platinum-based chemotherapy, PARP inhibitor, and delayed surgical resection.

## CASE PRESENTATION

**Fig. 1:**
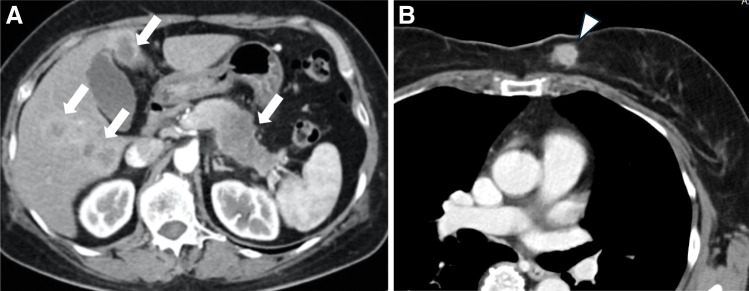
CT images. (**A**) Abdominal contrast-enhanced CT revealed a low-density mass occupying the entire pancreatic tail (white arrow) and multiple low-density masses in the liver (white arrows). (**B**) A 1.5-cm contrast-enhancing mass was identified in the lower inner quadrant of the left breast (white arrowhead).

**Fig. 2:**
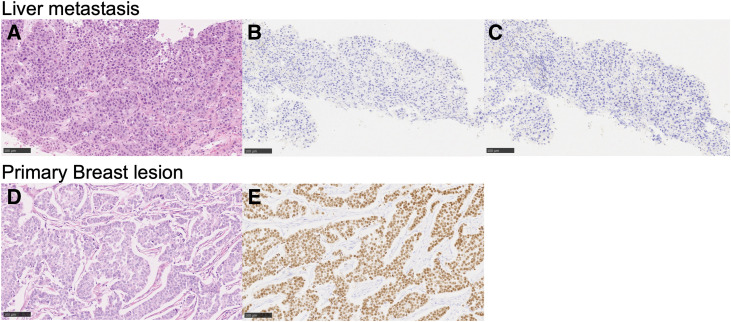
Histopathologic images. (**A**) H&E staining of the liver metastasis. (**B**) Immunohistochemical staining of the liver metastasis showing negativity for ER. (**C**) Immunohistochemical staining of the liver metastasis showing negativity for GATA3. (**D**) H&E staining of the primary breast lesion. (**E**) Immunohistochemical staining of the primary breast lesion showing positivity for ER. ER, estrogen receptor; H&E, hematoxylin and eosin

**Fig. 3:**
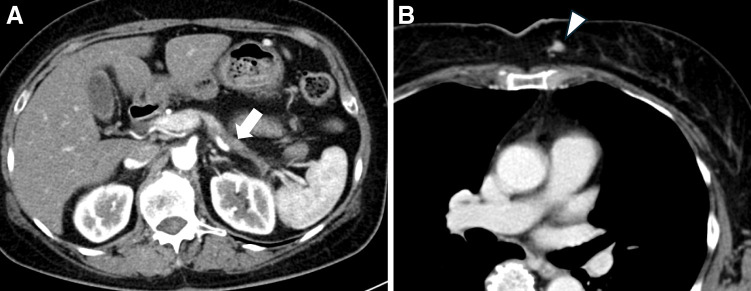
CT images. (**A**) Abdominal contrast-enhanced CT at 3 months after treatment initiation showed marked reduction of the pancreatic tail tumor (white arrow), with liver metastases undetectable on imaging. (**B**) The breast mass also showed a reduction in size (white arrowhead).

A woman in her 60s presented with a left breast mass and back pain. Her family history was notable for ovarian cancer in her mother, diagnosed at age 50. No other BRCA-associated malignancies were identified among first- to third-degree relatives. Imaging revealed a pancreatic tail tumor with multiple hepatic lesions suspicious for metastases and a 15-mm left breast lesion (**Fig. 1A**, **1B**). Liver biopsy confirmed metastatic pancreatic adenocarcinoma (cT3NxM1 (hepatic metastasis), stage IV); immunohistochemical staining was negative for ER and GATA3, arguing against breast origin (**Fig. 2A**–**2C**). Clinical staging of the left breast lesion based on imaging findings was cT1cN0M0, cStage I (UICC 8th edition). Tumor markers were markedly elevated (CEA 441 ng/mL; CA19-9 46933 U/mL). Based on multidisciplinary consensus, systemic therapy was prioritized, as pancreatic cancer was considered the primary determinant of prognosis; breast biopsy was therefore not performed at initial presentation. The breast lesion appeared to represent localized, nonmetastatic disease on imaging and was not considered to require immediate intervention. Furthermore, regardless of the biopsy result, the initial treatment priority would not have changed, as systemic therapy for metastatic pancreatic cancer appropriately took precedence over additional diagnostic procedures for the breast lesion at that time. Therefore, SOXIRI^6,7)^ was initiated, while the breast cancer was managed with careful observation. At 3 months after treatment initiation, a radiologic partial response was confirmed in both pancreatic and breast cancers (**Fig. 3A**, **3B**).

**Fig. 4:**
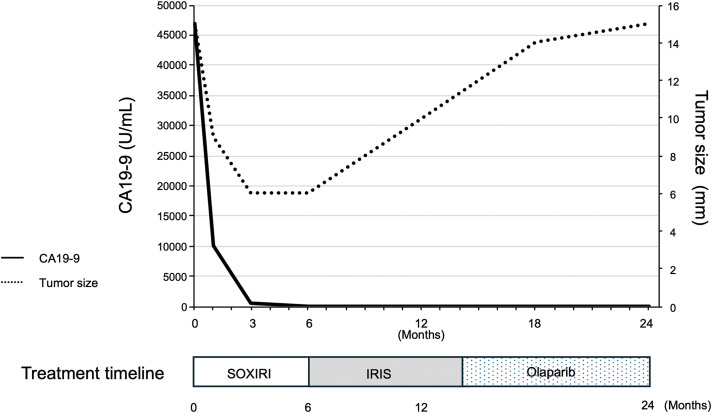
CA19-9 levels and breast tumor size over the first 24 months after treatment initiation. CA19-9 dramatically decreased from 46933 U/mL to 26 U/mL at month 6 following treatment initiation. The breast tumor initially responded to chemotherapy but gradually rebounded after oxaliplatin discontinuation, despite the pancreatic cancer remaining controlled on maintenance olaparib. CA19-9, carbohydrate antigen 19-9; IRIS, irinotecan and S-1; SOXIRI, S-1/oxaliplatin/irinotecan

SOXIRI was administered for 11 cycles, with a 20% dose reduction from the second cycle due to grade 3 diarrhea. Due to oxaliplatin hypersensitivity at 6 months after treatment initiation, treatment was continued with IRIS for 10 cycles after discontinuation of oxaliplatin; the administration interval was subsequently extended to once monthly due to cumulative fatigue. At this time point, tumor markers had completely normalized (CA19-9: 26 U/mL). Given the controlled status of the pancreatic cancer, a breast biopsy was performed to evaluate the breast lesion. The biopsy showed ER-positive (100%), progesterone receptor-positive (5%), human epidermal growth factor receptor 2 (HER2)-negative, and Ki-67 (25%) invasive ductal carcinoma, consistent with a hormone receptor (HR)-positive, HER2-negative clinicopathologic surrogate subtype (“Luminal type”) (**Fig. 2D**, **2E**). As this biopsy was performed after initiation of SOXIRI and after radiologic shrinkage of the breast lesion, this receptor profile may not fully reflect the pretreatment tumor biology. The ER positivity of the primary breast lesion, in contrast to the ER-negative liver metastasis, further supported the immunohistochemical distinction between the 2 malignancies. Germline testing identified a BRCA1 pathogenic variant. Genetic counseling was provided following disclosure of the result. Cascade testing was offered to at-risk family members; however, they declined. Approximately 1 year after treatment initiation, grade 3 hematologic toxicity associated with IRIS necessitated discontinuation of cytotoxic chemotherapy. Given the absence of disease progression, maintenance olaparib (300 mg twice daily) was subsequently introduced as an evidence-based maintenance strategy for BRCA-mutated metastatic pancreatic cancer, based on the POLO trial.^3)^ Five months after olaparib initiation, the dose was reduced to 250 mg twice daily due to grade 3 neutropenia. After achieving an excellent initial response to chemotherapy, the pancreatic cancer had maintained stable disease both on imaging and by tumor marker levels, whereas the breast tumor had gradually rebounded since oxaliplatin discontinuation (**Fig. 4**).

**Fig. 5:**
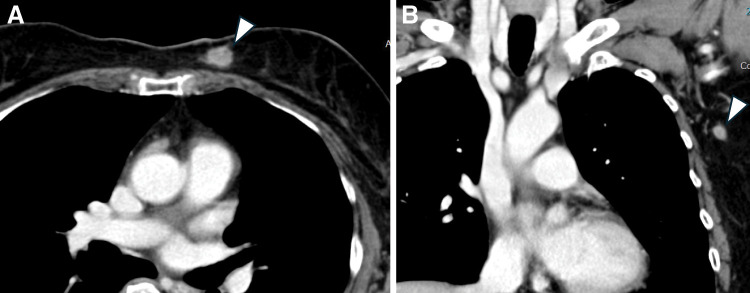
CT images. (**A**) Contrast-enhanced CT at approximately 28 months after treatment initiation showed an increase in breast mass size to 14 mm (white arrowhead). (**B**) An enlarged level I axillary lymph node was identified (white arrowhead), indicating progressive disease in the breast cancer.

Approximately 28 months after treatment initiation (about 16 months after olaparib initiation), the breast cancer progressed with axillary lymph node involvement (**Fig. 5A**, **5B**). Given durable systemic control of pancreatic cancer and preserved performance status, breast-conserving surgery with level II axillary lymph node dissection was performed. Although mastectomy, including contralateral prophylactic mastectomy, was discussed in accordance with guidelines for BRCA pathogenic variant carriers, breast-conserving surgery was selected based on the competing mortality risk posed by the metastatic pancreatic cancer and the patient’s strong preference for breast conservation. Pathological staging was pT2N1 (2/21), pStage IIB (UICC 8th edition), with the pathological tumor size exceeding preoperative imaging estimates, likely reflecting microscopic tumor extension beyond the imaging-apparent lesion. The 21-gene Oncotype DX Recurrence Score was high (RS 32), supporting the addition of adjuvant chemotherapy. Dose-dense epirubicin and cyclophosphamide (ddEC) was administered for 1 cycle; however, due to grade 3 neutropenia, the regimen was changed to conventional EC for 3 additional cycles, followed by weekly paclitaxel for 12 cycles. Adjuvant endocrine therapy with an aromatase inhibitor was subsequently initiated. Olaparib was discontinued during adjuvant chemotherapy for the breast cancer and was resumed thereafter to maintain control of the pancreatic cancer. Radiotherapy to the ipsilateral breast was omitted after multidisciplinary discussion considering competing mortality risk, cumulative systemic treatment burden, and patient preference.

At 77 months from treatment initiation, the patient remains alive without breast cancer recurrence or pancreatic cancer progression, while continuing maintenance therapy with olaparib and an aromatase inhibitor.

## DISCUSSION

This case highlights several important considerations regarding the surgical management of synchronous malignancies in patients with a germline BRCA1 pathogenic variant. Most notably, it challenges the traditional hierarchy in which stage IV pancreatic cancer uniformly precludes aggressive management of concurrent early breast cancer.

In the present case, sustained response to platinum-based chemotherapy followed by maintenance PARP inhibition resulted in long-term radiologic stability and normalization of tumor markers. Such durable systemic control suggests that selected patients with BRCA-associated metastatic pancreatic cancer may experience a clinical course distinct from historically reported outcomes.^3,8)^ When metastatic disease remains stable over an extended period, reassessment of oncologic priorities becomes essential.

The breast cancer, managed without definitive locoregional treatment, eventually demonstrated biological progression with axillary lymph node involvement. At that juncture, the breast malignancy represented a potentially curable disease whose natural history could compromise long-term survival if left unmanaged. Therefore, after confirming stability of metastatic pancreatic disease and preserved functional status, surgical intervention was considered appropriate. This adaptive surgical decision-making reflects longitudinal reassessment based on real-time disease behavior rather than initial staging alone.

In retrospect, the question of whether earlier surgical intervention for the breast cancer might have been appropriate warrants explicit consideration. At 3 months, a radiologic partial response had been confirmed in both malignancies; however, the durability of this response remained uncertain at that time point, and the risk of pancreatic cancer progression precluded commitment to elective breast surgery. At 6 months, CA19-9 had normalized and oxaliplatin was discontinued due to hypersensitivity; while pancreatic disease appeared controlled, the introduction of a less intensive regimen (IRIS) raised concern about potential disease destabilization, again making elective surgery difficult to justify. We continuously reassessed the feasibility of breast cancer resection throughout the observation period; however, the inherent unpredictability of stage IV pancreatic cancer behavior—even in BRCA-mutated cases^4,9)^—made it difficult to commit to elective surgery until durable systemic control had been established over an extended period. This case therefore illustrates not only the potential for long-term control in selected BRCA-associated pancreatic cancer patients but also highlights the challenge of prospectively identifying which patients will achieve such durable responses.

Interestingly, despite both tumors arising in the context of the same germline BRCA1 pathogenic variant, the pancreatic and breast cancers exhibited discordant responses during maintenance olaparib therapy. Whereas the pancreatic cancer remained controlled, the breast cancer progressed. This discordant response may be explained by tumor-specific genomic differences, such as biallelic BRCA1 inactivation in the pancreatic tumor, which has been associated with enhanced sensitivity to PARP inhibition, whereas the breast cancer may have retained heterozygosity or developed resistance mechanisms such as restoration of homologous recombination.^8,10–12)^ However, tumor-specific assessment of homologous recombination deficiency (HRD) status, loss of heterozygosity (LOH), and second-hit status was not performed in the present case. For surgeons and oncologists, this underscores the importance of evaluating each malignancy independently rather than assuming uniform therapeutic sensitivity.

While BRCA mutations are present in 2%–10% of all pancreatic cancer patients^13,14)^ and National Comprehensive Cancer Network guidelines recommend universal genetic testing, implementation in routine pancreatic cancer care remains insufficient. A Surveillance, Epidemiology, and End Results (SEER)-based study reported that only 5.6% of pancreatic cancer patients underwent germline genetic testing.^15)^ In the present case, BRCA testing was performed after treatment initiation, yet still proved valuable—retrospectively explaining the marked platinum sensitivity, prospectively enabling olaparib maintenance, and facilitating comprehensive genetic counseling for both the patient and her family. The profound therapeutic implications demonstrated here underscore the importance of genetic testing in all pancreatic cancer patients, ideally before treatment initiation to optimize therapeutic selection from the outset.

Historically, 5-year survival rates for pancreatic cancer in BRCA mutation carriers were reported to be 5% for BRCA1 and 4% for BRCA2.^16)^ Even with contemporary platinum-based chemotherapy and PARP inhibitor therapy, 5-year survival in stage IV pancreatic cancer remains rare; in the POLO trial, median overall survival was 19.0 months with olaparib maintenance versus 19.2 months with placebo, with no statistically significant difference between groups.^9)^ While Castro et al. reported the first case of synchronous breast and pancreatic cancer in a patient with a germline BRCA2 mutation,^17)^ survival outcomes were not described. To our knowledge, this is the first report demonstrating long-term (>5 years) survival with sustained control of both synchronous stage IV pancreatic cancer and operable breast cancer in a patient with a germline BRCA1 pathogenic variant managed with adaptive surgical decision-making. The present case, with 77 months of sustained disease control in both malignancies, represents an exceptionally rare clinical trajectory and provides practical insight into how molecularly targeted therapy may alter surgical sequencing strategies and enable exceptional long-term outcomes in selected patients with BRCA-associated multiple primary cancers. This case highlights that surgical timing in synchronous malignancies should be dynamically adapted based on longitudinal tumor behavior rather than fixed at initial diagnosis.

## CONCLUSIONS

In selected patients with BRCA-associated stage IV malignancy who achieve a durable response to platinum-based chemotherapy and PARP inhibitor maintenance, durable systemic control may create an opportunity for adaptive surgical decision-making regarding a concurrent operable malignancy, which may help prevent the breast cancer from becoming an additional threat to long-term prognosis. Dynamic treatment prioritization based on real-time individual tumor behavior, rather than initial staging alone, is essential in managing BRCA-associated multiple primary cancers.

## DECLARATIONS

### Funding

This research received no specific grant from any funding agency in the public, commercial, or not-for-profit sectors.

### Authors’ contributions

TA wrote the manuscript and performed a literature search.

MS reviewed and edited the manuscript.

TA and TY treated and observed the patient.

AY, TU, and RY performed the histopathologic and immunohistochemical evaluation and reviewed the relevant sections of the manuscript.

All authors reviewed and approved the final manuscript and agreed to be held accountable for all aspects of the research.

### Availability of data and materials

Data sharing is not applicable to this article as no datasets were generated or analyzed during the current study.

### Ethics approval and consent to participate

Ethics approval was waived by the Institutional Review Board of Nara Medical University for this single case report.

### Consent for publication

Informed consent to publish has been obtained.

### Competing Interests

The authors declare that they have no competing interests.
